# Free Space Detection Using Camera-LiDAR Fusion in a Bird’s Eye View Plane

**DOI:** 10.3390/s21227623

**Published:** 2021-11-17

**Authors:** Byeongjun Yu, Dongkyu Lee, Jae-Seol Lee, Seok-Cheol Kee

**Affiliations:** 1 Department of Smart Car Engineering, Chungbuk National University, Cheongju 28644, Korea; june9606@chungbuk.ac.kr (B.Y.); dlehdrb3909@chungbuk.ac.kr (D.L.); 2 Department of Control and Robot Engineering, Chungbuk National University, Cheongju 28644, Korea; js87@chungbuk.ac.kr; 3 Department of Intelligent Systems & Robotics, Chungbuk National University, Cheongju 28644, Korea

**Keywords:** autonomous vehicle, bird’s eye view transformation, convolutional neural network, heterogeneous sensor fusion, road detection, semantic segmentation

## Abstract

Although numerous road segmentation studies have utilized vision data, obtaining robust classification is still challenging due to vision sensor noise and target object deformation. Long-distance images are still problematic because of blur and low resolution, and these features make distinguishing roads from objects difficult. This study utilizes light detection and ranging (LiDAR), which generates information that camera images lack, such as distance, height, and intensity, as a reliable supplement to address this problem. In contrast to conventional approaches, additional domain transformation to a bird’s eye view space is executed to obtain long-range data with resolutions comparable to those of short-range data. This study proposes a convolutional neural network architecture that processes data transformed to a bird’s eye view plane. The network’s pathways are split into two parts to resolve calibration errors in the transformed image and point cloud. The network, which has modules that operate sequentially at various scaled dilated convolution rates, is designed to quickly and accurately handle a wide range of data. Comprehensive empirical studies using the Karlsruhe Institute of Technology and Toyota Technological Institute’s (KITTI’s) road detection benchmarks demonstrate that this study’s approach takes advantage of camera and LiDAR information, achieving robust road detection with short runtimes. Our result ranks 22nd in the KITTI’s leaderboard and shows real-time performance.

## 1. Introduction

Autonomous vehicles and advanced driver assistance systems (ADAS) are currently being improved. As computational capability progresses and markets grow, the autonomous vehicle is no longer an imaginary concept: it is slowly becoming a possibility. Free space detection, which classifies whether pixels are navigable by analyzing frontal environmental information with RGB color data and depth information, is essential in intelligent driving. Accurate detection is needed to guarantee safe driving and fast algorithm execution times and includes accurate sensor calibration and network outputs, especially when driving at high speeds.

Due to rapid developments in deep learning technology, efficient and novel feature extractors based on networks that solve semantic segmentation problems have been examined, and these can be applied to road segmentation [1,2]. Studies conducted by Chen et al. [2] and Yu and Koltun [3] overcame limited computer resources, extracted features at various scales, and made accurate semantic detections using large receptive fields. Along with accurate modules, various methods increased computation speeds by modifying the modules’ breadth or depth [1,4]. Moreover, several modules that enhance the expressiveness of their kernel’s robust features have been announced. These modules have revised convolutional and feature-extraction capabilities, both of which are used to create rich data [5,6,7]. Most methods that solve the free space detection problem use an image-based encoder–decoder structure [8,9]. Thanks to advances in semantic segmentation, numerous methods that distinguish obstacles from roads have been developed. However, single image data processing networks are strongly affected by illumination changes. They are not robust enough to filter out visual noise, such as blurry images and overexposure, producing incorrect results and lowering confidence in obtaining accurate detection results. Unlike cameras, which are passive sensors, LiDARs, which are active sensors, are more robust to changes in the surrounding environment and provide physical data, such as distance, height, and intensity, to complement visual data shortcomings. The stereo camera has physical data [10] that are similar to those that a LiDAR sensor has by computing a disparity map. Fan et al. [11] and Liu et al. [12] proposed stereo vision-based free space detection. Fan et al. [11] generated additional driving scene images and conducted semantic segmentation DCNN. Liu et al. [12] calculated a disparity map to obtain a vanishing line via a road surface model and detected road boundaries. However, the stereo vision method suffered from poor long-distance data that were more sensitive and unreliable than LiDAR.

RGB pixel data are combined with light detection and ranging (LiDAR) information that does not exist in a visual image to compensate for visual data shortcomings. LiDAR coordinates, as point cloud data, are projected onto the image plane during heterogeneous sensor calibration, which demands considerable operational time. Han et al. [13] and Caltagirone et al. [14] used camera pixel color data and LiDAR distances as inputs to a convolutional neural network (CNN) to segment free space. Additionally, Zhuang et al. [15] projected point cloud on image plane for 3D LiDAR semantic segmentation.

Aside from examining frontal data, transformed a data format has also been used. Caltagirone et al. [16] transformed unstructured point clouds into a bird’s eye view that was suitable for detecting roads with a CNN. Lu et al. [17], Roddick and Cipolla [18], and Wang et al. [19] utilized transformed bird’s eye view data. Roddick and Cipolla [18] predicted bird’s eye view maps directly from monocular images by incorporating a multiscale transformer pyramid to wrap image-based features to the bird’s eye view, and Wang et al. [19] transformed and fused features from different views of the LIDAR point cloud and images from cameras to detect 3D objects. This transformation method is able to effortlessly use geometric information from cameras to produce the best performance detector. Lee and Park [20] transformed a point cloud and an image into spherical coordinates to facilitate faster data transfer. Yang et al. [21] stitched images together to make an omnidirectional view and conducted panoramic semantic perception.

The main proposes of our study are listed below. We reduced the entire runtime and obtained a similar performance by changing the fusion method and convolution module and by adding some data preprocess:

- We employ a data-fusion method that quickly transforms a point cloud to a bird’s eye view rather than projecting it to an image plane, which requires more computational power. Here, the calibration time is reduced by projecting the data onto a plane instead of using a perspective transformation that multiplies several matrices such as sensor coordinate transformation, image plane projection, and undistortion.
- The data are RGB colors obtained from the transformed image and the z-axis measurements, point stack, and intensity [22]. The altitude difference [23] from the transformed point cloud is used as the network input by fusing camera and LiDAR data in the bird’s eye view plane.
- In addition to selecting an uncommon domain, improved modules are utilized to create a lightweight network with a large receptive field that can extract features efficiently [1,5].

In conclusion, the proposed system performs at a near real-time operating speed of over 37 FPS by applying quick calibration, obtains a MaxF of 94.89%, and ranks 22nd on the KITTI road benchmarks leaderboard.

## 2. Related Works

Free space detection presents an informative perception of the environment to autonomous vehicles. For several decades, research based on image processing has been expanding to address road segmentation problems. Ying et al. [1], Falola et al. [2], and Gao et al. [3] propose boundary detection-based approaches for identifying road regions. Approaches using region-based features have also been employed in free space detection. Various region-based road detection algorithms such as texture-based approaches [4,5] that identify textural differences between road and road-off regions and color-based approaches [6,7] that examine the characteristics of roads in color images have been developed.

Along with algorithms based on low-level features, many studies attempting to solve segmentation problems with deep CNNs have been carried out [8], and studies that considered free space problems to be semantic segmentation tasks have been conducted [8,9,10]. Similar to Fuse-Net [14], which combines geometric information from a LiDAR output with color data to tackle the segmentation problem, many studies using LiDAR for free space detection have been published. Nakagomi et al. [16], Gu et al. [17] and Caltagirone et al. [16] configured data from a single LiDAR to find navigable areas, and Han et al. [10] and Caltagirone et al. [14] fused images and point clouds to detect free space.

Algorithms for transformation from conventional frontal domains to other domains have been developed. For instance, Kühnl et al. [20] applied a bird’s eye view transform to streamline the complex scenarios of urban traffic, transformed point cloud [16] and image [17] data to a bird’s eye view as an input to a deep CNN, and developed a coordinate transformation from a Cartesian to a spherical model for the fast calibration of LiDARs and cameras [20].

Multisensor fusion algorithms can be divided into model-based white box and deep neural network (DNN)-based black box algorithms. The model-based method [1] processes sensor fusion by accumulating information about one object from multiple sensors. A Gaussian mixture finds the best measurement association and matches a single association from each sensor to make the fusion procedure more efficient. After the data association step, an unscented Kalman filter predicts object positions. The DNN-based method [2] takes advantage of DNN to sensor fusion. It selects intermediate fusion among early, late, and intermediate fusion candidates to achieve a more general, fast, and accurate prediction. A fusion block that exploits sensor information at each level and feeds the next block is proposed to fuse features from different sensors hierarchically.

**Figure 1:**
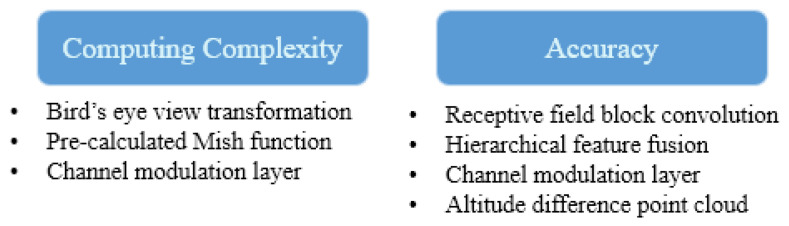
Lists of the proposed contribution of study.

In this paper, we propose a deep CNN-based system that obtains point clouds and images as inputs to detect free space, similar to other state-of-the-art (SOTA) methods. Furthermore, it transforms data into a bird’s eye view to reduce the calibration time and run time. Our transformation uses a rotation matrix based on homogeneous coordinates and a look-up table (LUT) to fuse the images and point clouds in the bird’s eye view. Further-more, minor calibration errors are resolved in a network layer. Figure 1 shows lists of the overall contributions of this paper in terms of computing complexity and accuracy.

## 3. Data Transformations

**Figure 2:**
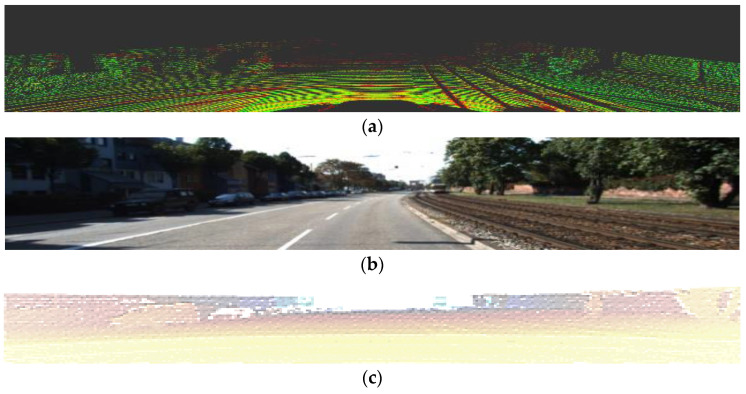
Examples of visual data. (**a**) The 3D point cloud raw data with 64-channel light detection and LiDAR data, (**b**) the 2D image with 1242 × 375 pixels, and (**c**) LiDAR data projected onto the image plane.

This section discusses a transformation method that projects a LiDAR point cloud onto a compatible set of 2D images, a format commonly used in CNNs for road detection. The proposed transformation method identifies the bird’s eye view coordinates that correspond to the 3D point cloud coordinates. Figure 2c is an example of a point cloud projected onto an image plane.

### 3.1. Perspective Transformation

Perspective transformation converts a point cloud in the LiDAR coordinates to pixel data coordinates in an image plane using intrinsic and extrinsic parameters [14]. Doing so fuses the camera data and the LiDAR data. In other words, a transformation matrix is applied to a 3D point $p_{l}={\left(x,\;y,\;z,\;1\right)}^{T}$ and is matched with a 2D pixel $p_{i}={\left(u,\;v,\;1\right)}^{T}$. In Equation (1), the transformation matrix rotation and the translation are T, the rectification matrix is R, and the camera-intrinsic matrix is K. Here, the $p_{i}$ coincident with the projected $p_{l}$ is expressed as:

(1) $$\lambda \;p_{i}=K\;R\;T\;p_{l}$$

where λ is a scaling factor. T rotates and translates the LiDAR coordinates to camera coordinates and can be expressed as:

(2) $$T=\left[\begin{array}{cccc} r_{00} & r_{01} & r_{02} & t_{x}\\ r_{10} & r_{11} & r_{12} & t_{y}\\ r_{20} & r_{21} & r_{22} & t_{z}\\ 0 & 0 & 0 & 1 \end{array}\right],$$

where $r_{ij}$ and $t_{k}$ are rotation parameters (roll, pitch, and yaw) and translation parameters, respectively. R transforms coordinates to normalized coordinates, and K projects them to pixel coordinates. These variables are expressed as:

(3) $$R=\;\left[\begin{array}{cccc} 1 & 0 & 0 & 0\\ 0 & 1 & 0 & 0\\ 0 & 0 & 1 & 0 \end{array}\right],\;K=\;\left[\begin{array}{ccc} f_{x} & 0 & c_{x}\\ 0 & f_{y} & c_{y}\\ 0 & 0 & 1 \end{array}\right],$$

where *f* and *c* represent the lens focal length and principal pixel point, respectively. The matrices in (1) project vast amounts of points, nearly 100 k per frame, from LiDAR into a uniform image container. The LiDAR geometric data are then in the image’s format, and the camera’s color information along with the LiDAR range data are input to a CNN to detect free space.

### 3.2. Bird’s Eye View Transformation

Bird’s eye view transformation is a method that changes an image’s orientation to where it is parallel to the ground. Because the data need to be rotated and translated, the image coordinates are transformed into normalized coordinates. Equation (4) shows the transformation matrix that changes $p_{i}={\left(u,\;v,\;1\right)}^{T}$ in the image coordinates to $p_{n}={\left(X_{n},\;Y_{n},\;1\right)}^{T}$ in the normalized coordinates, where K is the camera-intrinsic matrix from Equation (3).

(4) $$p_{n}=K^{-1}p_{i},$$

After the coordinate system changes, the T matrix, similar to Equation (2), reconfigures $p_{n}$ to $p_{u}={\left(X_{u},\;Y_{u},\;1\right)}^{T}$ in the normalized bird’s eye view coordinates to adjust the axis to those coordinates. Finally, $p_{u}$ is projected onto the bird’s eye view plane, $p_{b}={\left(x,\;y,\;1\right)}^{T}$, by adjusting the scale. Equation (5) is the projection formula:

(5) $$\lambda \;p_{b}=\left[\begin{array}{ccc} S_{x} & 0 & c_{tx}\\ 0 & S_{y} & c_{ty}\\ 0 & 0 & 1 \end{array}\right]p_{u},$$

where *λ* is the scaling factor, $S_{\left(x,\;y\right)}$ is the image height ratio, and $c_{\left(tx,\;ty\right)}$ is the principal point in the bird’s eye view plane.

**Figure 3:**
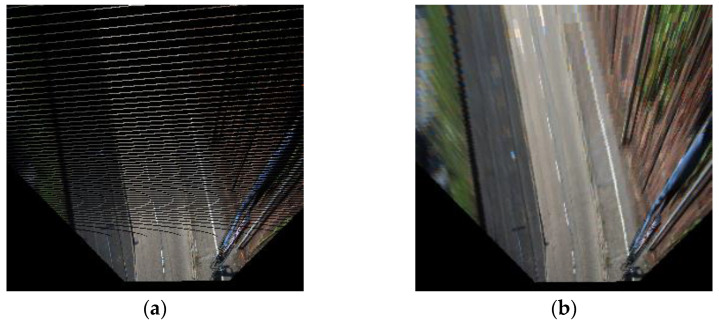
Different transformation methods. (**a**) Data transformed from image coordinates to bird’s eye view coordinates and (**b**) data transformed from bird’s eye view coordinates to image coordinates.

However, the transformation comes with an aliasing issue caused by insufficient discrete data, so an inverse transformation is used to convert the coordinates for anti-aliasing to address this issue. Figure 3 shows an example of the transformation issue and the anti-aliasing transformation.

As opposed to the previous procedure, the inverse transformation finds a point $p_{i}={\left(u,\;v,\;1\right)}^{T}$ that is homologous with $p_{b}={\left(x,\;y,\;1\right)}^{T}$ from the image and bird’s eye view coordinates, respectively. The pixel $p_{i}$ can be found from $p_{b}$ by multiplying it with the inverse of the matrix that projects the data to the bird’s eye view plane. The matrix R rotates and translates the coordinates’ axes, and the intrinsic matrix K projects the image coordinates from the normalized coordinates. Since both pre- and post-transformed data were filled into a structured data container (the image), transforming $p_{b}$ to $p_{i}$ is a function. Here, the bird’s eye view image is the domain, and the original image is the codomain. Therefore, instead of performing a time-consuming set of matrix multiplications, a lookup table (LUT) can be used to obtain transformations faster. The table stores precalculated transformations from $p_{i}$ to $p_{b}$, so it does not perform matrix multiplications for every transformation but projects color data by simply building a coordinate correspondence map.

**Figure 4:**
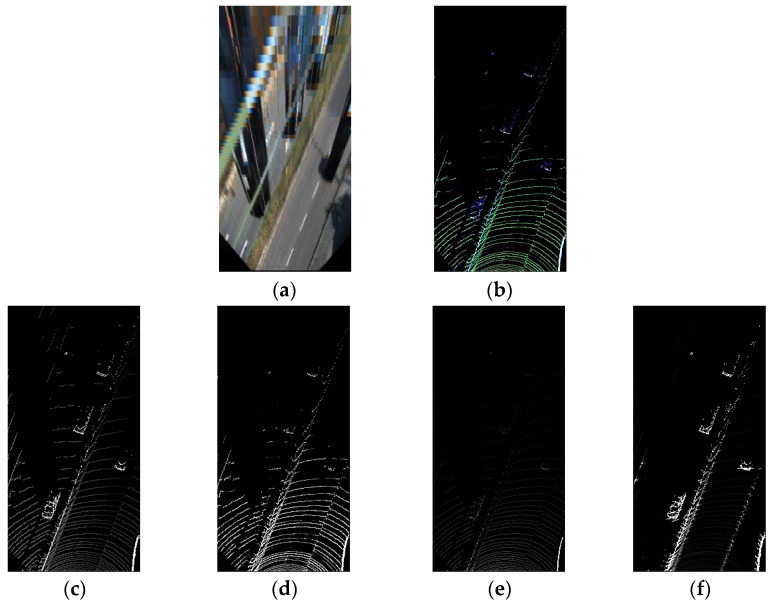
Examples of data transformed to the bird’s eye view. (**a**) Image and (**b**) point cloud, ignoring 4th channels. The bottom row shows examples of each channel’s point cloud data in grayscale, (**c**) max height value in a cell, (**d**) max intensity value in a cell, (**e**) number of points in a cell, and (**f**) altitude difference from surrounding cells.

The transformed point cloud geometric information combined with a transformed image is used as an input to the CNN. Two steps are needed to transform the unstructured 3D data point cloud into structured 2D data suitable for CNN input. First, a cell with measurements corresponding to one pixel is constructed to project the point cloud’s *x*–*y* plane onto the 2D image plane. The cell size is set to 0.1×0.1 m and is projected from a 20 m wide y∈[−10,10] m by s 40 m long x∈[6,46] m cell into 200×400 pixels. Next, four sets of channel data, including the maximum height, the number of points, maximum cell intensity, and altitude difference from surrounding cells, are inserted in the image format. The transformed data are shown in Figure 4.

## 4. Bird’s Eye View Free Space Detection

**Figure 5:**
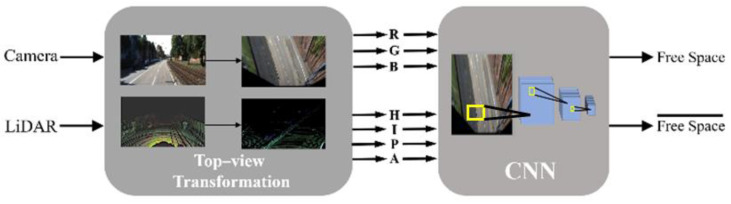
Overall system architecture. RGB stands for the image’s color information. H, I, P, and A represent the maximum height, maximum intensity, number of points, and altitude difference, respectively. The left-hand images in the first block show their original format, and the right-hand images show their transformed format.

Figure 5 shows the overall system architecture including data transformation and road prediction. The first box, labelled as Top-view Transformation, takes visual data and preprocess them, as noted in Section 3. Additionally, the second box, labelled CNN, predicts free space using the proposed network and will be discussed in this section. For a given transformed RGB image (^t^I) and projected point cloud (^t^L) generated by projecting both the image and the LiDAR points to a bird’s eye view plane with extrinsic parameters, the proposed system targets free space (F)

The additional ^t^L data help the system divide curbstones or objects from the road to segment it using information such as the height or intensity. This section discusses a system that has been designed to detect free space. Section 4.1 describes an efficient receptive field pyramid (ERFP) module, which makes the model lightweight and accurate. Section 4.2 describes an encoder–decoder-based multipath network composed of ERFP modules. Section 4.3 depicts the data augmentation methods employed for their robustness when handling distortions.

### 4.1. Efficient Receptive Field Pyramid Module

**Figure 6:**
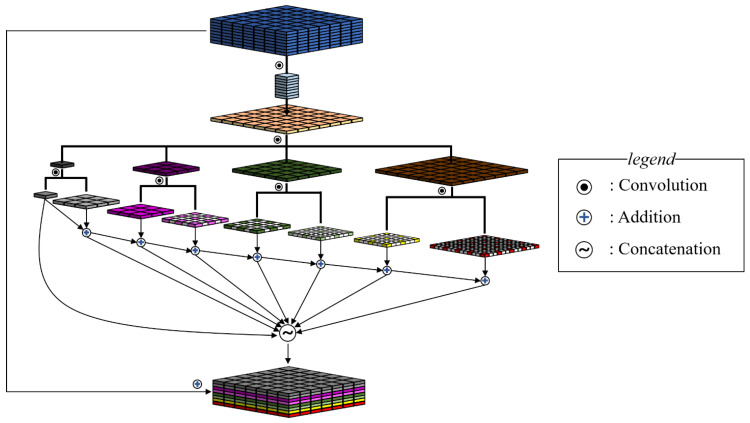
Schematic flow chart for the proposed module’s efficient receptive field pyramid (ERFP). After a feature map’s depth is fed as input and the depth is reduced to D/P divided by the number of pyramids P using pointwise convolution, standard convolutions are applied with various kernel sizes. Dilated convolutions with dilation rates proportional to the kernel size are used to extract differing scales. Using the pyramid made by collecting P latent feature maps, whose depth D/P * with expansion rate E is applied with hierarchical feature fusion (HFF), the output feature map with depth D * E is formed.

An example of an ERFP module is depicted in Figure 6. ERFP is a method that organizes a spatial pyramid of varied scales that have been extracted from a single feature map [2,5,6]. The ERFP module that populates the network is a factorized form of a spatial pyramid with a pointwise convolution layer that modulates several channels for quick operation.

Pointwise convolution is a (1×1) convolution that reduces the computational parameters to increase a network’s speed. A conventional spatial pyramid takes the input feature map $F_{in}$ ∈ ${\mathbb{R}}^{\left(C_{in}\times H\times W\right)}$ and outputs $F_{out}$ ∈ ${\mathbb{R}}^{\left(C_{out}\times H\times W\right)}$ using ***P*** dilated convolutions with (k×k)-sized kernels and dilation rates given by $2^{p-1},$ (p =1, 2, 3, ⋯, P). Here, H and W are the feature map’s height and width, and $C_{in}$ and $C_{out}$ are the feature map’s channels. Hence, the original pyramid has learnable $k^{2}\cdot C_{in}$ · $C_{out}\cdot$***P*** parameters to produce an output feature map. The ERFP module exploits the parameter ***P***, the stacked dilated convolution number, to reduce the number of parameters. After applying a pointwise convolution that reduces the input feature’s depth by 1/***P***, there are $\frac{k^{2}\cdot C_{in}\cdot C_{out}+{C_{in}}^{2}}{P}$ parameters when stacking the ***P*** pyramid with dilation convolution using the $\frac{{C_{in}}^{2}}{P}$ parameters of the input’s reducing channel and the output’s $\frac{k^{2}\cdot C_{in}\cdot C_{out}}{P}$ parameters from the downsized input feature map. Pointwise convolution reduces the parameters by a factor of $\frac{k^{2}\cdot P^{2}\cdot C_{in}\cdot C_{out}}{k^{2}\cdot C_{in}\cdot C_{out}+{C_{in}}^{2}}$. For one of the hyperparameters employed in the network, ***P*** = 8, *k* = 3, $C_{in}$ = 32, and $C_{out}$ = 64, so this study’s system has 60.6 times fewer parameters than a conventional spatial pyramid does.

The next layers include standard convolutions with multi-sized kernels that assemble differing feature scales and dilated convolutions with dilation rates in proportion to their size, which allows a large receptive field and shortened computation. Thanks to the channel modulation layer reducing the entire computational cost, a pair of dilated convolutions from each standard convolution is additionally supplemented to create richer representations. By combining these layers, including the depth modulating convolution, standard convolution, and dilated convolution, ERFP can cover a wide region with varied scales using small operating quantities.

The pyramid of various scale features is the layer that extracts more varied scale features than extractions from a single scale input. The input, which shrinks to 1/***P*** of its channel, was sampled into the varied scaled information using a different sized receptive field of standard convolutions. ***P***/2 standard convolutions with the kernel size (k×k), where *k* = $2^{q-1},$ $\left(q=1,\;2,\;3,\;\cdots ,\;\frac{P}{2}\right)$, splits the reduced input into ***P***/2 branches with differing extent information. After this, dilated convolution with a dilation rate proportional to the receptive field is applied to obtain information at different scales from the same sized input and is composed of features at varied distances. Two dilation rates in each branch’s dilated convolutions are used to create a ***P*** feature pyramid from the ***P***/2 branches.

This study utilizes a method that combines hierarchical feature fusion (HFF) and concatenation to merge feature pyramids. HFF is a merging method that adds feature maps with larger receptive fields more frequently than maps with smaller receptive fields. This technique resolves the checkerboard problem and takes advantage of a large receptive field without losing the starting pixel’s characteristics due to a weighted value that decreases as the distance increases. The latent feature maps, whose depth is treated as $C_{out}$/***P***, are sequenced for the output feature pyramid. Its sequential output is connected to the input feature map to take advantage of the skip layer’s strengths.

### 4.2. Structure of the Network

**Figure 7:**
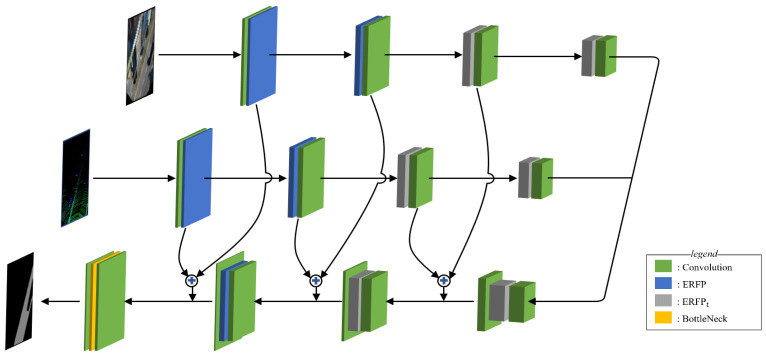
Structure of the network. The proposed network is based on an encoder–decoder formation that has split encoding paths. The image and point cloud are encoded individually and are processed in a flattened decoder to detect free roads. The encoded image and a point cloud at the same encoder level are combined and linked to the decoding path to compose the skip layer.

Figure 7 shows the proposed network with its encoder–decoder-based structure. Its feature-extracting encoder contains encoding path L, which explores the point cloud features, and encoding path I, which investigates the image’s salient features. There are three reasons to go through two independent encoder paths instead of concatenating the input point cloud and image during the first step. First, the encoder paths are split to align location errors caused by inaccurately calibrating the image and point cloud while transforming their data into a bird’s eye view in the feature map’s latent layer

Through the feature compression encoding process and answer extraction decoding process, convolution kernels learn parameters to calculate properly weighted sums that not only detect roads but also handle calibration errors, and two symmetric branches are used to enhance adjustment, such as CalibNet [24]. Second, this approach avoids nullifying point cloud features with sparse density. Third, the robust characteristics of the image and point cloud are different, allowing the differentiated paths to encode dissimilar features. After encoding, the decoding process detects free space by upscaling the reduced feature map to its original size. Pixel-Shuffle is used to upscale the feature map during the decoding process [25]. It uses depth information to provide width information, which separates the object’s outline more clearly. It utilizes the encoder’s same-level features to provide a shortcut to obtain longer gradient paths as the feature map becomes more upsampled while allowing the network to learn from various gradients. Features from I and L are added and concatenated with same-sized decoder features as an input for the next decoding layer. This skip layer is applied at every level where the feature map size is the same for the encoder and decoder.

In addition, the ERFP_t_ module, which has a relatively small receptive field, is used at the levels with feature maps smaller than a quarter of their original size for both the encoder and the decoder. The ERFP_t_ module utilizes fewer standard convolutions with different sizes to lessen the number of pyramids that is generated while creating two feature maps per branch. It lessens memory usage and computations by conducting the convolution operation less frequently.

### 4.3. Data Augmentation and Learning Details

This subsection explores data augmentation, which yields diverse training datasets through distortion, and the learning details used for obtaining optimal network parameters. Because the transformed data that are being utilized as the network’s input has similar distributions of close and distant pixels, it is vulnerable to the distortion that caused by tall objects and slopes. Most notably, it takes advantage of geometric augmentation, which is resistant to noise generated from inclinations.

**Figure 8:**
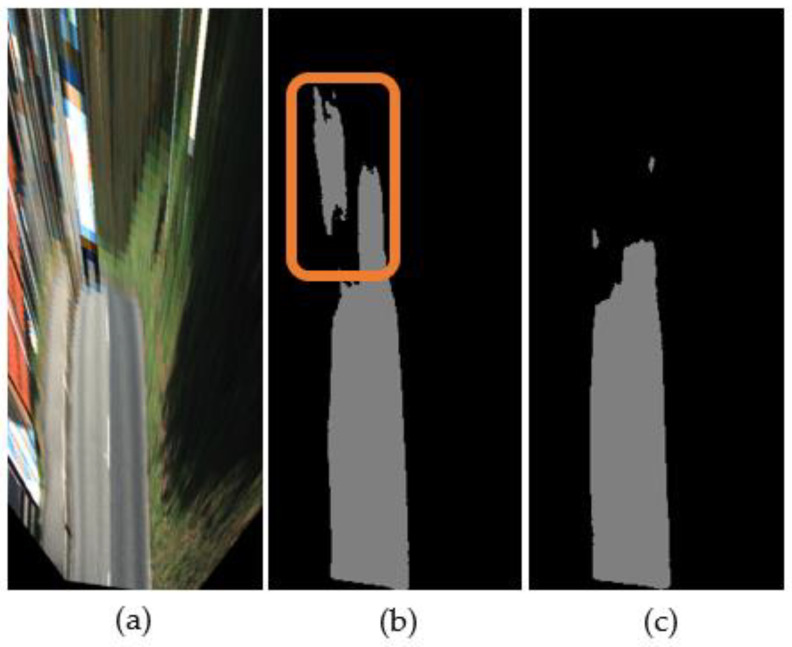
Examples of results with and without data augmentation. (**a**) Input image; (**b**) a result of training without augmentation; (**c**) a result of training with augmentation.

Figure 8 shows examples of failed and successful cases. Augmentations such as scale augmentation (adjusts the bird’s eye view transformation’s height), incline augmentation (controls the road angle by adjusting the rotation matrix’s pitch), and 3D augmentation (modifies various axes) support the ability to distinguish transformed objects.

The training was conducted on 2000 epochs using the Adam optimization algorithm with a learning rate of 0.0001 [26]. After reaching 1000 epochs, the learning rate was set to decrease to 1/10 over 1000 iterations exponentially, and the weight decay was set to 0.0001. A mish function was used as an activation function in the network, which was defined as x·tanh(softplus(x)), where $softplus\left(x\right)=\ln\left(1+e^{x}\right)\;$ [27]. Through many properties such as the unbounded positive domain, bounded negative domain, non-monotonic shape, and smooth derivative, mish reduced our training time and provided performance in this experiment. This function computed and saved the Jacobian matrices of all of the layers in order to propagate learning. These matrix multiplications are an inefficient computational process from a memory usage perspective, as they increase the network’s GPU memory share. Therefore, to increase the GPU’s memory efficiency, the mish function’s derivative, $f^{\prime }\left(x\right)=\tanh\left(softplus\left(x\right)\right)+x\cdot sigmoid\left(x\right)\cdot \mathrm{sech}{\left(softplus\left(x\right)\right)}^{2}$, where f(x) is the mish function, was calculated in advance and was implemented by diving the forward and backward sections for network learning. The Algorithm 1 that shows the function’s efficient application is provided in this study.
**Algorithm 1:** Implementation of the memory efficient mishctx: stashed information for backward computation; input: data to be applied to the mish; grad_output: gradient to the precious layer. **Class** mish **is** **Function** forward(ctx, input) **is** ctx ← input **return** input * tanh(softplus(input))  **Function** backward(ctx, grad_output) **is**  x ← ctx  sigmoidX = sigmoid(x)  softplusX = softplus(x)  tanhX = tanh(softplusX)  sechX = 1/cosh(softplusX)  **return** grad_output * (tanhX + x * sigmoidX * sechX^2^)

## 5. Experiments

**Figure 9:**
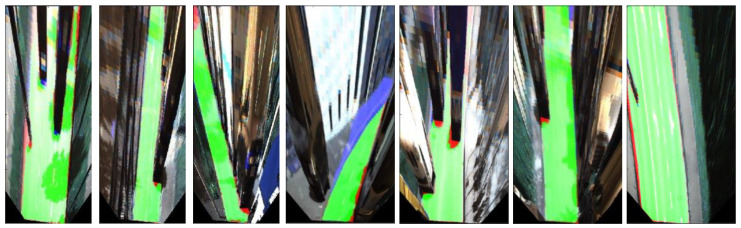
Examples of the road detection result projected onto a bird’s eye view image plane. The green pixels indicate true positives, red pixels indicate false negatives, and blue pixels indicate false positives.

The Karlsruhe Institute of Technology and Toyota Technological Institute (KITTI) dataset was used to test the system through experiments [28]. The KITTI dataset consists of point cloud data in a perspective coordinate system from a 64-channel LiDAR, Velodyne HDL-64E. The road detection sector provides 1242 × 375-pixel RGB images that have been synchronized with the point cloud. In addition to the visual data, camera-based intrinsic and extrinsic parameters are included for calibration. Performance comparisons of sensor configurations and comparisons between the bird’s eye view transformations and image plane projection times demonstrated the system’s efficiency. The KITTI road benchmark test compared the proposed method to SOTA methods. The computer specifications were as follows: an NVIDIA RTX 3090 GPU, Intel core i9-10900X CPU, and Ubuntu 18.04 OS. We used Python and PyTorch to build the proposed network, which consumed 195 MB GPU memory for the inference, and the proposed network had 7.5 MB parameters. Some examples of the road detection results projected onto the image are presented in Figure 9.

Figure 9 shows some successful cases and corner cases of proposed method. This figure shows some inappropriate results from when the height value of the point cloud was changing slowly, as in the case of a curved road or when the vehicle shadows are long. However, in most cases, it can be seen that our method is robust enough for severely bad environment such as traffic jam, small objects such as people or cyclists, and rail roads.

### 5.1. Performance Comparison by Sensor Configuration

Table 1 depicts the ablation experiment results and shows the advantages of using additional point cloud geometric information with the RGB images. The KITTI training dataset is composed of 95 urban marked (UM) images, 96 urban multiple marked (UMM) images, and 98 urban unmarked (UU) images. One piece of data per ten images from the training dataset was added to the test scenario validation dataset. The first environment described the performance of the network that only used ^t^I. The next scenario conducted a performance test using only ^t^L. Afterward, the proposed method using both ^t^I and ^t^L was evaluated. Because the encoding paths of the first and second cases were cut in half, the encoder’s depth was doubled to compensate for the diminished encoder. F1 measurements, average precision, precision, and recall were compared to show performance differences based on sensor configurations, and all measures were assessed using ground truth data in the transformed format. The table indicates that configurations using LiDAR have higher scores in recall but lower scores in precision than the camera-only configuration. This is because the network frequently predicts the area outside of the road, such as a corner curb or uphill road, as free space when geometric information is missing. This tendency increases precision by making excessively large guesses regarding the road boundary. However, considering the overall measurement, the results indicate that a heterogeneous sensor configuration is better than single-sensor configurations.

### 5.2. Comparison of the Transformation Time

An LUT was utilized to transfer an image to a bird’s eye view plane, and the system eliminated its z-axis to project a point cloud that calculated the cells’ maximum height, maximum intensity, stashed points, and altitude difference. Since the image was already contained in the image plane, image transformation occurred instantaneously. Point cloud transformation occurred by multiplying three different matrices to project a 3D={x, y, z} point to a 2D={u, v} pixel in the image plane. Table 2 shows the domain transformation times. During testing, the total time to project a bird’s eye view image was 10.94 ms, approximately 13 times faster than the time needed to transform point clouds into an image plane (146.48 ms).

### 5.3. KITTI Road Benchmark

Table 3 compares the proposed algorithm’s benchmark with other existing methods. An urban scenario, including all of the categories, was assigned as the test dataset. In addition to the evaluation measures in Table 1 (MaxF, AP, PRE, and REC), the runtime was utilized to analyze the method’s speed–performance ratio. Some SOTA algorithms were compared to the study’s proposed method.

Some SOTA algorithms were compared to the study’s proposed method. Here, RBANet [29], OFANet [32], and HA-DeepLab [10] used a single sensor and camera. Likewise, ChipNet [31] and LoDNN [16] only used LiDAR in their proposed system. ChipNet preprocessed point cloud data by organizing them in a spherical view, as a LiDAR naturally scans, and LoDNN transforms the point cloud into the bird’s eye view plane. On the other hand, PLARD [23], NIM-RTFNet [30], and LidCamNet [14] take advantage of multisensor data. They transform a 3D point cloud into the image plane by projecting its LiDAR coordinates. Runtimes for all of the methods only included the prediction time without the data transformation time. However, the proposed method’s runtime was treated as the sum of its prediction time and transformation time. When comparing the proposed method with other SOTA algorithms using the evaluation measures, the proposed method was approximately 1.46% less accurate than heavier networks such as PLARD and RBANet, which are placed above our proposed method in Table 3. However, it was almost 4.8 times faster even though the domain transfer time was added to the network prediction time. Furthermore, compared to the lighter methods that are ranked below our proposed method in Table 3, such as HA-DeepLab, the study’s system showed a 0.72% improvement while running 1.2 times faster. The bird’s eye view plane transformation to fuse data ensures that the calibration time is extremely fast, and the channel modulation layer streamlines the convolution module so that it improves the computing cost and execution time of the network. After reducing the computational complexity, the HFF method that combines feature maps with higher weights that are closer to the origin and that improved the structure that populates the plural dilated the convolution layers from a standard convolution to diversify the gradient path. This enabled the robustness and accuracy of the system to improve while maintaining the runtime. As a result, the proposed algorithm segmented roads at almost real-time speeds, faster than other light methods and slightly less accurately than other heavy methods that have demonstrated SOTA performance.

## 6. Conclusions

In this study, a camera–LiDAR fusion-based CNN architecture was developed to perform bird’s eye view road detection. Common methods demand massive computational costs because they conduct several matrix multiplication computations to transform amorphous point clouds. In the proposed method, applying a small LUT computation to transfer standardized images to the bird’s eye view plane and eliminating the point cloud’s z-axis created data configurations that were 10 times faster than other methods. The transformed image and point cloud were used as independent encoder inputs to align and extract dissimilar features and were up-sampled to the original size using the decoder. In this process, an ERFP with a large receptive field and strong kernel expressiveness was used. Some of the limitations of our proposed method, such as inaccurate results at a long distance and low-resolution data as a result of data transformation, still remain and degrade the performance. We think that these issues can be solved by considering the dynamic kernel size of the convolution according to the distance and deep-learning data processing. The designed road detection system is powerful, ranking 22nd in the KITTI benchmark, and is very efficient, taking only 27 ms to perform all of the tasks.

## Figures and Tables

**Table 1:** Performance evaluations according to the sensor configuration. Best scores are highlighted in bold.

	MaxF (%)	AP (%)	PRE (%)	REC (%)	Runtime (s/Frame)
Camera	94.31	92.16	**98.31**	90.63	**0.024**
LiDAR	94.36	92.17	96.68	92.15	**0.024**
Camera + LiDAR	**94.91**	**92.88**	96.39	**93.48**	0.025

**Table 2:** Results of calculating the transformation time to each plane.

Data Format	Transformation Time (ms/Frame)	
	Bird’s Eye View Plane	Image Plane
Image	0.46	0
Point cloud	10.48	146.48

**Table 3:** KITTI road benchmarks compared to other methods.

	MaxF (%)	AP (%)	PRE (%)	REC (%)	Runtime (ms/Frame)	Operating Complexity
PLARD [23]	97.03	94.03	97.19	96.88	160	Heavier network than ours
RBANet [29]	96.30	89.72	95.14	97.50	160	
LidCamNet [14]	96.03	93.93	96.23	95.83	150	
NIM-RTFNet [30]	96.02	94.01	96.43	95.62	50	
**Study method (BJN)**	**94.89**	**90.63**	**96.14**	**93.67**	**27**	
HA-DeepLab [10]	94.83	93.24	94.77	94.89	60	Lighter network than ours
LoDNN [16]	94.07	920.3	92.81	95.37	18	
ChipNet [31]	94.05	88.59	93.57	94.53	12	
OFANet [32]	93.74	85.37	90.36	97.38	40	

## Data Availability

http://www.cvlibs.net/datasets/kitti/eval_road.php.
